# Stevens-Johnson Syndrome–Like Reaction After Exposure to Pembrolizumab and Recombinant Zoster Vaccine in a Patient With Metastatic Lung Cancer

**DOI:** 10.1177/2324709620914796

**Published:** 2020-03-24

**Authors:** Ivy Riano, Cagney Cristancho, Thomas Treadwell

**Affiliations:** 1 MetroWest Medical Center, Framingham, MA, USA; 2 Boston University, Boston, MA, USA

**Keywords:** Stevens-Johnson syndrome, pembrolizumab, recombinant zoster vaccine, skin reactions, skin cytotoxicity, mucocutaneous adverse drug reactions

## Abstract

Stevens-Johnson syndrome (SJS) is a life-threating mucocutaneous reaction predominantly induced by drugs. Targeted cancer therapies such as pembrolizumab, which has been approved for the treatment of metastatic malignancy, can cause severe skin toxicities, including SJS. They are rare and inconsistently reported. In this article, we report the case of a 80-year-old woman with metastatic non–small cell lung cancer who had a SJS-like eruption involving oral mucosa after 15 weeks of exposure of pembrolizumab (6 doses) and 7 days after 1 dose of recombinant zoster vaccine. SJS is a rare blistering disorder with high mortality rate and significant morbidity. Causes include drugs, herpes viruses, and immunization. The timing of the eruption soon after the receipt of recombinant zoster vaccine suggests a role of vaccination in our patient, yet patients receiving cancer immunotherapy may develop late-onset skin toxicity. Therefore, we recommend long-term monitoring for mucocutaneous reactions after initiation of pembrolizumab. Further research is needed to characterize the immunological pathogenesis and improve timely recognition and treatment strategies.

## Introduction

Stevens-Johnson syndrome (SJS) is a severe mucocutaneous reaction usually caused by drugs.^1^ Pembrolizumab, an anti-programmed death-1 (anti-PD-1) antibody, is approved as therapy for several metastatic cancers. SJS has become a rare serious complication associated with immunotherapy for cancer.^2^ Very few cases of SJS associated with pembrolizumab have been reported in the literature.^3^ Recombinant zoster vaccine (RZV), an alternative to the live-attenuated herpes simplex vaccine, was recently approved for prevention of herpes zoster. There is no report of RZV as a cause of SJS. In this article, we present the case of a patient with metastatic non–small cell lung cancer who had a SJS-like eruption involving oral mucosa after 6 doses of therapy with pembrolizumab and 1 dose of RZV. The patient’s lesions improved after prednisone treatment and cessation of pembrolizumab.

## Case Presentation

An 80-year-old Caucasian woman had a 13-year history of lung adenocarcinoma. She was treated with lobectomy. Despite several surgeries and chemotherapy, progression to advanced lung adenocarcinoma with invasion of the visceral pleura occurred. Based on biomarker testing (PD-L1 positive, EGFR/ALK negative), the patient received immunotherapy with a single-agent pembrolizumab that resulted in improvement of disease progression. During this treatment period, she received RZV. The patient presented with a 2-day history of multiple small oral ulcers. She had last taken pembrolizumab 2 days prior and the first dose of RZV 7 days before presentation. She received a total of 6 doses of pembrolizumab before presentation. Associated symptoms included fatigue. There were no other new medications recorded either previous experience with other immunotherapy agents or corticosteroids. The oral mucositis was considered nonspecific; thus, an antiseptic solution was prescribed. After 2 days, she developed new ulcers in the tongue associated with difficulty swallowing solids. She received treatment with acyclovir without improvement. Subsequently, the patient exhibited worsening of ulcers over the lips and a nonpruritic and nontender rash in the upper back and upper extremities.

**Figure 1.**
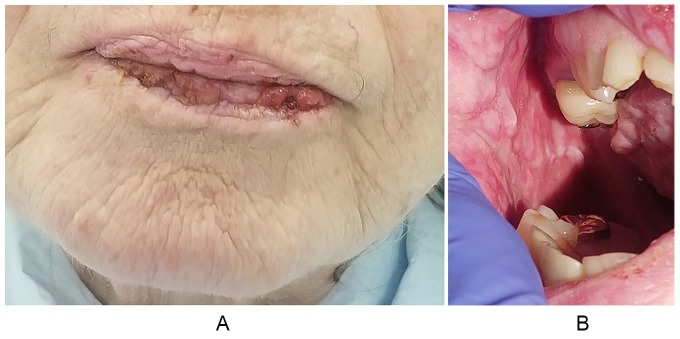
Clinical features. (A) Hemorrhagic crust more prominent in the lower lip. (B) Buccal mucosa with extensive vesicular lesions with erythematous borders.

The physical examination revealed hemorrhagic crust more prominent in the lower lip, showing cracking and fissuring with blood encrustation (Figure 1A). Oral mucosa examination showed extensive vesicles with erythematous borders scattered in hard palate, mucosa, and gums (Figure 1B). The tongue was denuded, with prominent taste buds and a rough surface appearance. Some scabs were noticed in the upper extremities and upper back without any surrounding erythema.

**Figure 2.**
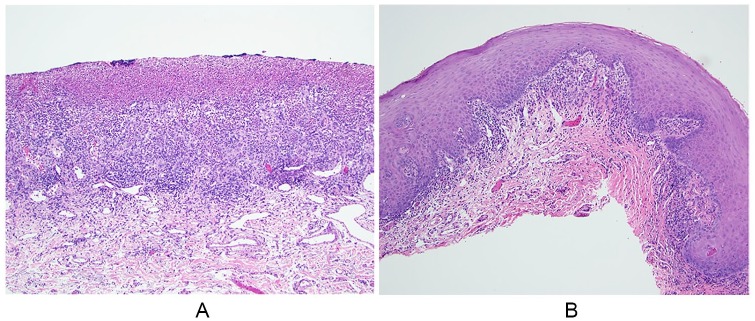
Histopathologic findings. (A) Ulcer bed composed of granulation tissue and covered by a fibrin clot. A diffuse acute and chronic inflammatory cell infiltrate is appreciated throughout the subjacent connective tissue stroma (hematoxylin and eosin [H&E]; 100×). (B) Mildly parakeratotic stratified squamous epithelium overlying fibrous connective tissue. A patchy predominately chronic inflammatory cell infiltrate is noticed, focally aligned along the epithelial-connective tissue interface (H&E; 100×).

A buccal mucosa biopsy revealed focally ulcerated mildly parakeratotic stratified squamous epithelium overlying fibrovascular connective tissue. The ulcer bed was covered by a fibrin clot composed of enmeshed erythrocytes, neutrophils, and lymphocytes (Figure 2A). Numerous ectatic endothelial-lined vascular channels were noticed throughout the subjacent connective tissue stroma, which exhibits a diffuse acute and chronic inflammatory cell infiltrate (Figure 2B). No immunoreactants (C3, immunoglobulin [Ig] G, IgA, IgM) were detected.

The viral culture and polymerase chain reaction did not detect herpes simplex virus. Although the histopathologic findings were nonspecific, when correlated with the clinical presentation and onset of new medications, the findings were compatible with the diagnosis of SJS-like eruption. The patient received prednisone, which slowed the progression of her lesions. She had not been maintained on steroid therapy prior. The patient did not receive the second dose of RZV and pembrolizumab was discontinued. At a 1-month follow-up appointment, her initial eruptions improved. Unfortunately, the patient subsequently presented to our hospital with severe sepsis from a perforated duodenal ulcer. It is possible that the corticosteroids given for her SJS-like syndrome contributed to her death.

## Discussion

Stevens-Johnson syndrome is recognized as a severe delayed-type hypersensitivity reaction that is almost always caused by drugs. SJS is a blistering disorder that involves mucosa and cutaneous tissue. It is characterized by <10% of total body surface area of epidermal detachment; when more than 30% of skin is involved the disorder is called toxic epidermal necrolysis.^4^ Clinically, SJS presents with a prodromal period of flu-like symptoms (fever, malaise, anorexia) followed by erythematous dusky-red macules that can involve neck, trunk, and extremities as well as inflammation and pain of oral, ocular, and genital mucosa.^1^ Although SJS is rare, the mortality rate approaches 30% with significant short- and long-term morbidities.^1^ The most common drugs associated with SJS are sulfonamide antibiotics, anticonvulsants, nonsteroidal anti-inflammatory drugs, and corticosteroids. Other etiologies, such as viruses (ie, herpes simplex virus) and vaccinations, have been implicated as a cause of SJS.^1,5^ Some predisposing factors, namely, drug-specific T-cell-mediated cytotoxicity, and genetic susceptibility of the patient (human leukocyte antigen [HLA] and non-HLA genes) may play a role in its pathogenesis.^1,6^ The immune response in SJS is mediated by T-cell activation by drug-related haptens.^7^ In recent years, a new concept has been elucidated describing a direct and reversible interaction of the drug between T-cell receptors and major histocompatibility complex molecules to stimulate cytokine secretion and cytotoxicity.^7^

Our patient developed SJS-like eruptions in oral mucosa after 15 weeks of exposure of pembrolizumab (6 doses) and 7 days after 1 dose of RZV. SJS only rarely occurs 8 weeks following suspected drug exposure, but late onset in patients receiving immunotherapy has been reported.^6,8^ The association of some anticancer drugs with rare life-threatening serious adverse events such as SJS has been anecdotally reported, but has not been systematically examined.^9^

Pembrolizumab is usually a well-tolerated therapy for metastatic cancers. The most common immune-related skin lesions reported are lichenoid reaction, eczema, and vitiligo.^10^ Pembrolizumab rarely causes skin reactions, but severe responses such as SJS can occur. The latent period between pembrolizumab exposure and onset of symptoms in SJS varies from 7 to 140 days.^8^ In histologic analyses of adverse cutaneous induced by anti-PD-1 therapy, there is evidence of accumulation of CD8+ T-cell at the dermoepidermal junction and CD8+ T-cell exocytosis into the epidermis with apoptotic keratinocytes.^2^ These features are also observed in SJS.^2^ In addition, gene expression profiles from skin lesions caused by anti-PD-1 therapy and SJS has similarities.^2,8^ These findings suggests that anti-PD-1 antibody can induce SJS-like adverse reactions.^2^

Furthermore, cases of SJS following herpes zoster vaccination are exceedingly rare. In a recent systematic review, there were 2 postmarketing surveillance studies implicating live-attenuated varicella vaccine with SJS. A total of 8 patients between 1 and 29 years old were reported with a latency period of 3 to 5 days.^11^ The immunologic theory behind vaccine-induced cutaneous hypersensitivity states that antigens in the vaccine are expressed on the surface of keratinocytes, generating a CD8+ T lymphocyte immune response against epidermal cells. This leads to apoptosis of keratinocytes and detachment of the dermal-epidermal junction.^11^ Our patient received the new vaccine RZV that promotes strong CD4+ T-cell and humoral immune response against recombinant proteins. SJS induced by RZV has not been reported.^12^

What caused our patient’s SJS-like reaction? It is difficult to ignore the appearance of her severe mucocutaneous eruption shortly after she received zoster vaccine, suggesting that herpes virus antigens, perhaps in combination with her immunotherapy, may have induced the cytotoxic skin reaction. However, immunotherapies require time to induce immune responses, and skin immune–mediated adverse effect may take longer to appear compared with cytotoxic therapies.

Timely diagnosis can facilitate cessation of a suspected drug, leading to decreased morbidity from associated drug reactions. We recommend long-term monitoring for mucocutaneous adverse drug reactions after initiation of pembrolizumab. More investigations of severe skin toxicity associated with immunotherapy are warranted to elucidate the immunological pathogenesis as well as improve early recognition and treatment strategies.
